# Race biology

**DOI:** 10.1186/s41065-020-00161-x

**Published:** 2020-11-25

**Authors:** Anssi Saura

**Affiliations:** Department of Molecular Biology, Umeå University, SE-901 87 Umeå, Sweden

**Keywords:** anthropometrics, eugenics, sterilization, Hereditas

## Abstract

**Background:**

The founders of Hereditas envisioned that race biology would be a major subject that had social applications with utmost importance in the near future. Anthropometrics was in this context understood to be the pure and eugenics the applied science. Sweden had a long tradition in physical anthropometry. Herman Lundborg, member of the advisory board of Hereditas, united the anthropometric and eugenic approaches in a synthesis. He was the first head of the Institute for Race Biology in Sweden. The contents of Hereditas reflect the development of race biology in the Nordic countries.

**Conclusions:**

The initial enthusiasm for applied race biology did not last long. In the 1920’s Hereditas carried papers on both physical anthropology and eugenics. Most paper dealt, however, with human genetics without eugenic content. Two papers, published in 1921 and 1939 show how the intellectual climate had changed from positive to negative. Finally only human genetics prevailed as the legitimate study of the *human race* or humankind. A belated defense of eugenics published in 1951 did not help; geneticists had abandoned anthropometrics for good around the year 1940 and eugenics about a decade later. In spite of that, eugenic legislation was amended astonishingly late, in the 1970’s. The development was essentially similar in all Nordic countries.

## Background

A *race* is a population distinguishable from other populations by hereditary phenotypic characters. Members of a human race may freely produce offspring with representatives of other races. Even if race has been socially constructed [1], it has been an immensely powerful construction that has shaped the lives of millions. Restrictions based on geography, skin color, language, culture and religion have been imposed to maintain groups of people as endogamous units. The Indian caste system may have been the most extreme effort of social control of human reproduction [2]. Segregation in the American south, apartheid in South Africa, aristocratic societies and prejudices represent less brazen attempts of social control. The Universal Declaration of Human Rights was drafted to abolish the above practices.

*Race biology* stands for biological anthropology with eugenics as an applied approach. In the Nordic countries the two make up a temporal sequence so that anthropological measurements came first, followed with the social aspect.

Linnaeus had in *Systema naturae* divided humanity into four differentiated groups using geographic but otherwise arbitrary criteria. A systematic research on race was begun in Sweden in the 1840’s. The father of anthropometrics is Anders Rezius (1796–1869), who took measures on Sámi (Lapps) [3, 4] . He introduced craniometry, the study of skull dimensions, and coined terms such as dolichocephalic and brachycephalic, long- and short-skulled, respectively. His work led to a revision of race classification in Europe. Gustaf Retzius (1842–1919) followed his father as an anthropologist. He was interested in the three races of Sweden: Nordic, Finnish and Sámi (Lapps). The Nordics were in general dolichocephalic, the Sámi brachycephalic and the Finns intermediate between the two. Typical Sámi were seen to have a short stature and dark hair [5], while the Swedes and Finns were tall and had fair hair. Retzius jr. was, in particular, keen to measure Finnish skulls, [6]. The giant volume titled *Anthropologia Suecica* [7] sums up the accumulated results of 60 years. In retrospect, it is difficult to make out why this immoderate compilation of data was collected. It was certainly used to promote ideas of the pre-eminence of the “superior dolicocephalic race” e.g. [8].

Given this alleged superiority of the Nordic race, positive measures had to be taken not only to preserve it at the degree of purity then attained and, if feasible, to improve the positive and desirable and weed out the negative characteristics. Herman Lundborg extended the work of Retzius and Fürst [7] into a medical dimension. He [9] defended in 1903 a doctoral dissertation on myoclonus epilepsy or Unverricht-Lundborg disease. Lundborg used pedigrees extracted from church records to find out the lines of descent and the transmission of the disease. His thesis represented a new and useful approach in the study of heritable diseases. Lundborg was rewarded with accolades including the prize of the Swedish Medical Society. The recognition encouraged him to extend the study beyond strict human genetics. In a follow-up study comprising an extended family with 2232 members Lundborg [10] not only demonstrated the inheritance of the epilepsy but, in addition, controversial evidence for degeneration that expressed itself as alcoholism and debauchery. Cousin marriages had exacerbated the misfortune. Lundborg recommended that eugenic measures should be applied to cure the afflicted family. The result was a giant volume (35.5 × 28× 5.5 cm, 740 pages). In addition to his own data, he described the racial characters of the Swedish people following Retzius and Fürst [10].

The Swedish Society for Race Biology (*Svenska Sällskapet för Rashygien*) was established in the autumn of 1909 in Stockholm. *Rashygien,*“race hygiene”, is a German and Nordic synonym for eugenics, albeit with some anti-Semitic overtones [11]. The society had a lofty aim: “to support efforts to improve the health of the body and mind in the future generations”. The evolutionary biologist Wilhelm Leche was the chairman and the Nobel Prize winner (chemistry) Svante Arrhenius the most prominent member of the executive board. Herman Lundborg emerged as an active advocate for race biology. Together with the zoologist Nils von Hofsten, the psychiatrist Elis Essen-Möller and the plant breeder Herman Nilsson-Ehle he created a network to promote eugenic consciousness [12, 13]. The race biologists set out publishing popular booklets to educate the public and began lobbying for a national institute for race biology. The painter Anders Zorn financed the Ethnic Type Fair (*Rastypsutställningen*), a collection of photos and paintings showing Swedes together with Sámi, deviant types and criminals. The explanatory text included the elements of genetics. Starting from Stockholm and Uppsala, the exhibition toured several towns in 1919. It was an all-time success that attracted more than 50% of the adult population in many cities [14]. That is no wonder: Anders Zorn specialized in painting nude blond maidens displaying their charms in lush lakeside etc. settings.

### Hereditas

Herman Nilsson-Ehle, the plant breeder of international fame, was the driving force in the founding of the Mendelian Society in Lund in 1910 and, ten years later, in setting up its journal *Hereditas*. He was involved in virtually everything that was done to further the progress of genetics in Sweden between the years 1910 and 1935 [15–17]. To secure a financial base for a genetics journal the Mendelian Society sent an appeal to Swedish patrons of science and culture. The tone of the appeal, signed by Herman Nilsson-Ehle, president, and Carl August Hallqvist, secretary general, was patriotic. They stressed the significance of genetics in plant breeding and applied race biology; the plant breeding institutions of Svalöf and Weibullsholm were taken up by name. Race biology has social applications that will be of utmost importance in the near future. If diseases and traits are found to have a genetic basis, then the society should respond through appropriate measures. Financial utility is less important than the control of asocial behavior etc. Evidently a critical and strictly scientific treatment is needed here [16, 17].

Herman Lundborg was elected member of the advisory board of Hereditas from almost the very beginning. Hereditas was envisioned to be a publishing forum for Nordic geneticists so that Swedes, and authors of PhD theses in particular, were given priority, but if space allowed, contributions from other Nordic countries were included.

### Applied race biology

Theodore M Porter [18] has pointed out that physicians and state officials tasked with overseeing insane asylums throughout the nineteenth century attempted to understand the nature and origins of madness. They were the precursors of pedigree research and eugenics.

In the first half of the twentieth century eugenics was a legitimate branch of applied genetics. *Negative eugenics* strove to lower the frequency of detrimental traits. Individuals expressing them could be counseled to refrain from marrying. As an alternative, they could be sterilized. To protect the society at large, mentally disabled persons or criminals could be sterilized or castrated against their will. *Positive eugenics* recommended various incentives to help the genetic elite to reproduce at least as fast as those less endowed.

Sterilization of the feeble-minded, epileptics and asocial elements was a major goal of negative eugenics. Even though the evidence that the traits underlying these characteristics were hereditary was often scant, the Nordic countries made in the 1930’s laws allowing the sterilization of the undesirables. The course of events was essentially similar in each case, so that a short overview will suffice here. The German occupation of Norway gave rise to an interlude in this development, the most astonishing feature of which was that the laws were repealed as late as in the 1970’s. I shall give a short overview of race biology and eugenics in Denmark, Finland, Iceland, Norway and Sweden.

#### Denmark

The anthropological committee (*Den antropologiske Komité*) was founded in 1905. The board members included the police physician Søren Hansen, the pioneer geneticist Wilhelm Johannsen, the statistician Harald Westergaard and the psychiatrist August Wimmer [19]. The committee was a private society that obtained public funding in 1920. It lobbied for eugenic legislation. Marriage restrictions were the first step in 1909–1913. In 1912 Søren Hansen was elected member of the Permanent International Eugenics Committee in London. Karl Kristian Steincke, a social democrat and minister of justice in the 1920’s, formulated a race hygiene program for the welfare state as a part of social reform. The control of reproduction was seen as a prerequisite to finance the welfare state. His proposal 1920 “The welfare state of the future” had eugenics as an integral part of social reform. It was based on Darwinism, Mendelism and the degeneration hypothesis of the French psychiatrist Bénédict Augustin Morel [11].

The Danish Sterilization Commission of 1924 had three representatives of the Anthropological Committee, including Johannsen. He criticized the proceedings on theoretical, not practical grounds, pointing out that e.g. alcoholism is not hereditary. The Commission finished its job in 1926 and the preliminary sterilization law of 1929 was based on its work. It passed the parliament with flying colors. Only the conservatives were skeptical. The preliminary law was replaced in 1934 and 1935 through a new one that made compulsory sterilization possible for the mentally retarded until 1967 and for criminals until 1972 [11, 20].

#### Finland

The positive social aspect came first in Finland. The emphasis was preserving and furthering a language [21]. The majority of Finns spoke Finnish while Swedish was the mother tongue of an influential minority. In 1911 Jenny Florin donated 100000 Finnish marks to the Swedish Literary Society in Finland (*Svenska Litteratursällskapet*) on the condition that the interest of the fund would be used to promote medical science. The Society decided that the interest of the fund would be used for “a comprehensive scientific examination of the mental and physical health of the Swedish-speaking population in Finland and of all circumstances, which might exercise influence on it. It was particularly pointed out that the significance of heredity for the health of the people should be made a special object of research” [22].

Ossian Schauman and Jarl Hagelstam, professors of internal medicine and neurology, set with the zoologist Harry Federley up a committee (*Florinska kommissionen*) to carry out the program outlined above. The committee screened the occurrence of mental diseases, tuberculosis etc. In addition, anthropological measures, housing conditions and nutritional status were recorded. The anthropological survey was motivated through the success of the ethnic type fair in Sweden, but it was soon abandoned. The pilot phase covered about 23.000 persons. In addition, prizes were awarded to mothers with a healthy family background and at least four healthy and strong children between the ages of 4 and 14 years [22].

Public Health in Swedish Finland (*Samfundet Folkhälsan i Svenska Finland*) was established in 1921 to promote this program of positive eugenics. Schauman was the first chairman, followed by Federley, by then the professor of genetics. The other members of the executive board were professors of medicine and philanthropists. *Folkhälsan* created a network of community nurses to combat child mortality and campaigned against alcohol abuse, smoking and tuberculosis [21, 22].

In 1929 the government of Finland set up a committee to investigate whether compulsory sterilization of the feeble minded etc. would be desirable. They had in mind the Danish preliminary law of 1929. The sterilization law passed the parliament in 1935. It became, however, soon apparent that the law was seldom enforced. Consequently, a new and stricter law was put into effect in 1951. It was repealed in 1970 [23, 24]. Kajanoja [25] sums up the anthropological studies made on Finns and related peoples.

#### Iceland

The professor of medicine Gudmundur Hannesson took anthropologic measures on the Icelanders [26]. A sterilization and abortion law was passed in 1937 in Iceland, the last one among the Nordic countries. People diagnosed as feebleminded could be sterilized against their consent [27]. The law was amended in 1975 [28].

#### Norway

The pharmacist Jon Alfred Mjøen prescribed eugenics to cure disorders of the health of mind and body. He was politically affiliated with the liberal Venstre party. In 1906 Mjøen not only founded and financed Winderen Biological Laboratory (*Winderen Biologiske Laboratorium*) in Kristiania (Oslo) to study the effects of race crossing, i.e. between the Sámi and Nordic Norwegians and the inheritance of intellectual capacity. He saw the Yellow Peril, i.e. a takeover of East Asians, as an impending threat. Mjøen had contacts with Alfred Ploetz, who founded in 1906 The German eugenics society (*Deutsche Gesellschaft für Rassenhygienie*). In 1908 Mjøen initiated the Norwegian eugenics program (*Racebiologi og Fortplantninsgshygiene*). In 1912 The Permanent International Eugenics Committee in London used it as a model. Mjøen was appointed as a self-evident member to the committee. He advocated negative measures such as the permanent segregation and sterilization of the unfit as well as positive ones such as maternal insurance and propaganda for health and wholesome food along with prophylactic treatments to protect the unborn [29, 30].

In 1914 Mjøen published a treatise titled Racie hygiene, (*Racehygiene*, [31]). The university scientists Kristine Bonnevie and Otto Lous Mohr attacked his ideas as genetically unsound. Mohr had analyzed child mortality in relation to population increase (emigration from Norway was intense at that time). He criticized Mjøen’s view that western nations are dying because of an increase of bad elements and advocated responsible birth control, which will lead to an automatic optimal breeding program. The animal breeder Christian Wriedt criticized Mjøen as well. Bonnevie and Mohr founded in 1919 *Norsk forening for arvelighetsforskning* (the Norwegian Society for Genetic Research) and kept Mjøen out. Undaunted, Mjøen initiated the Norwegian Consultative Committee for Racial Hygiene (*Den norske konsultative komiteen for rasehygiene*). The antagonism between Mjøen and the university geneticists was, in part, motivated through a competition for the meager financing available. Mjøen carried, however, the day: his ideas were used as groundwork for eugenic legislation [30, 32].

In 1919 Mjøen founded the journal The Nordic Race (*Den Nordiske Race*). It published papers mostly in Scandinavian languages. J.A. Mjøen was the editor-in-chief.and the publisher Winderen Laboratorium at Oslo; it was printed in Copenhagen. The editorial board included an impressive list of luminaries such as Leonard Darwin, Charles Benedict Davenport, Herman Nilsson-Ehle, Henry Fairfield Osborn, Alfred Ploetz and Hans Virchow.

The Norwegian parliament passed in 1934 almost unanimously a law that allowed legal sterilization on eugenic and social grounds. Compulsory sterilization was allowed only in cases of severe mental deficiency. In 1942 the Nazi puppet Quisling government pushed through a sterilization act. It followed the severe German model and remained in force until the liberation of Norway in May 1945. The number of mental patients sterilized following the introduction of the act rose at first sharply but evidently never reached the goal the Germans had envisioned. The original law of the year 1934 was abolished in 1977 [33].

#### Sweden

The Swedish Society for Race Biology had lobbied for the establishment of a Nobel Institute for Racial Biology at the Karolinska Institute in Stockholm. These attempts failed, but the Swedish Parliament founded the Swedish State Institute for Racial Biology (*Statens Institut för Rasbiologi*) in Uppsala in 1921 with Herman Lundborg as its director [13, 34]. The proposition to the Parliament to found the Institute had been enthusiastically supported by virtually all politicians, including Hjalmar Branting and Arvid Lindman, leaders of the Social Democratic and Conservative parties, respectively. The Society published a jubilee volume [35] to commemorate the event. The book contains short entries on anthropology, eugenics, cytology and genetics in Sweden and Finland.

The Uppsala Institute opened its doors 1 January 1922 with Lundborg as its head. It was then the only race biology establishment in the world financed by a government [36] . It served as the model for The Kaiser Wilhelm-Institut für Anthropologie, menschliche Erblehre und Eugenik founded in 1927 in Berlin. The Swedish Society for Race Biology and the State Institute remained the only organizations of their kind in the Nordic countries. In 1925 the Institute organized a Nordic meeting on race biology and anthropology that was attended by some 30 participants [36].

Herman Lundborg was the director of the State Institute from 1922 until 1935. He was mainly interested in race biology and published several popular booklets on the danger of degeneration. He argued that fecundity was high in northern Sweden, i.e. a low-income area where race mixing was prevalent [37]. Olsson [38] had in 1918 used a very extensive data set covering all of Sweden to refute this argument. He showed that the correlation between fecundity with income was positive; the middle class reproduced faster than the lower one.

Lundborg’s first *magnum opus* (quite literally, it shared the giant format of Retzius & Fürst 1902 [7] and Lundborg [10]) and was titled “The racial characters of the Swedish nation” [34]; there was also an abridged version in German [39]. The book contains, in addition to extensive statistics, wonderfully naïve photos. It is written in a self-adulatory tone that is difficult to take seriously today. It is no mean feat: the material is made up of 47,387 men between 20 and 22 years old, some 50% of all males in Sweden of that age cohort. Lundborg gave out a popular version with maps in Swedish [40], intended to be used in schools. In the above books Lundborg refers positively to the ideas of Nordic supremacy proposed by the linguist Rolf Nordenstreng (Nordenstreng [41]. The Institute for Racial Biology in Uppsala took measures on some 100.000 people, mostly military conscripts and minorities.

Herman Lundborg shared with Anders and Gustaf Retzius the interest in measuring the Sámi. Lundborg & Wahlund [42] is a massive treatise on Sámi in the Retzius-Lundborg tradition. Like Lundborg & Linders [34] it was well received. To mention an example, the Dutch colonial authorities purchased six copies to Batavia (Jakarta). It was followed by Dahlberg & Wahlund [43] that contained the photos and accessory material. The latter had the bulky format of the former, but it is much thinner. Here the authors repudiate physical ethnological race biology as obsolete pseudoscience in no uncertain words. The tenets of anthropological race biology had begun to crumble on a wide front around 1934, e.g. [44, 45]. Lundborg had retired embittered in 1935 and Gunnar Dahlberg was his successor as the head of the Institute for Race Biology. Lundborg’s swan song was *Västerlandet i fara* “The West in peril” [46], a demagogic booklet.

The zoologist Nils von Hofsten, a board member of the Institute of Racial Biology, reviewed in the capacity of a scientific expert on the State Medical Board (*Socialstyrelsen*) the applications for sterilization that were made legal through the law passed in 1934 until 1953. He reviewed and expanded the law of 1934 so that a new law of 1941 was mainly his making. His credentials as a geneticist are meager. He felt that it was the responsibility of the society to cut out the dead wood of the “rot in the stock” [13]. The sterilization law was repealed in 1976.

A eugenicist streak had been present in the socialist movement already in the early 1900’s. Arthur Engberg, a social democrat and newspaper editor had supported eugenics and anti-Semitism in the 1910–20’s but in the capacity of the minister of education and research he cut the financing of the State Institute in 1933. Engberg saw that Lundborg’s constant study trips to Lapland were a waste of money [47, 48]. The social democratic politician and (university chancellor) Östen Undén supported Gunnar Dahlberg as the successor of Lundborg instead of the eugenically oriented Torsten Sjögren [17].

Dahlberg had in 1922 started his career as Lundborg’s assistant at the Institute for Racial Biology. He oversaw the change of the institute from one of ethnology to human genetics. Dahlberg published e.g. on alcoholism and diseases as a social problem and debunked doctrines of race in popular books such as Dahlberg [49]. In particular “Race reason and rubbish” [50] was widely read and translated into several languages. It contains the modern view in a nutshell. Dahlberg was one of the 23 signatories of the eugenics manifesto of 1939 titled “Social Biology and Population Improvement” [51], a proclamation signed by leading geneticists 3 days before the onset of World War II. This public declaration condemns racism but maintains a somewhat ambivalent attitude towards the social aspects of eugenics.

### Race biology in Hereditas

As stated above, Hereditas was envisioned to cover applied race biology, in particular aspects deemed to be “of utmost importance in the near future”. Robert Larsson, the first editor-in-chief was an ardent supporter of eugenics [13, 17]. How did Hereditas carry into effect these expectations? The following review covers the contributions to the subject, from race biology to human genetics from the first issue of 1920 up to the end of race biology in the early 1950’s. Let us take one author at a time and in chronological order. This allows us to see how the authors changed their views on a controversial subject that underwent a drastic paradigm change in the span of 30 years.

#### Herman Lundborg

In 1920 Herman Lundborg was elected member of the advisory board of Hereditas. He came out with a paper on “The hereditary transmission of genotypical deaf-mutism” in the first issue of the first volume, the second paper ever published in the journal [52]. Lundborg had consulted the eminent human geneticist Wilhelm Weinberg in selecting a proper proband method [53]. Lundborg had another paper in the first volume as well [54]. Using data on the incidence of tuberculosis in Sweden he developed an argument that the offspring of race (i.e. Swede, Finn or Sámi) crosses are more susceptible to tuberculosis than the population at large. He used the plant breeding work of Oscar Hagem and Herman Nilsson-Ehle as supporting evidence. In addition to northern Sweden, where the Swedes, Finns and Sámi interbreed, he showed that levels of TBC in Stockholm and Gothenburg were as high as in the North. These big cities had attracted people from all parts of Sweden and from abroad, with race mixing as a result. The line of reasoning is roughly as follows: each race has developed a specific genetic resistance to TBC. The genetic control of susceptibility is polygenic, organized in coadapted gene complexes in race. In race crosses the resistance breaks down. The rest of the paper is a routine description of eye and hair color in the three races. Interestingly, he found out that red hair is associated with diseases and criminal behavior. He drew conclusions on the basis of raw data without the benefit of a statistical analysis.

Lundborg [55] developed the above hypothesis on the deleterious effects of interbreeding between races further. He started from the observation that people with a mixed parentage are taller but argued that they are disease-prone. Mixing races results in a chaotic genetic constitution “Genchaos” with an unbalanced phenotype as a consequence. Here he has extended the study to cover the entire European continent. His examples cover Italians, Germans and Russians.

He [56] measured eye and hair color, and cepthalic and facial indices in the population of the northernmost part of Sweden. The Swedes and Finns had light eyes and fair hair, while the Sámi had dark hues. The Swedes and the Sámi had extremes in head dimensions with the Finns intermediate between the two. There had been gene flow from the Sámi to the Finns; when the former abandon their nomadic way of life, they adopt Finnish customs.

Lundborg’s ideas represent mainstream race biology of the 1920’s. E.g. Jon Alfred Mjøen and others voiced similar concerns [57]. – In addition to the above papers that we now may consider to represent rather unrestrained scientific racism, Lundborg had a short report [58] on the sex-linked inheritance of ichthyosis simplex. The condition is characterized with rough and scaly skin; of the multiple forms of the disease this is the mildest one. Lundborg used church records to demonstrate how this form of ichthyosis is inherited.

#### Emanuel Bergman

In the first volume of Hereditas the Swedish physician Bergman described a family with hereditary tremor [59]. This is pure human genetics.

#### Halfdan Bryn

Bryn was a military physician and anthropologist in Trøndelag in central Norway. In the first volume of Hereditas Bryn [60] published the results of extensive observations on eye color and cephalic index in Selbu and Tydal, two nearby but isolated municipalities. He tried to find out the inheritance of these above traits and did not discuss the results in an anthropological context. Later on, he may have been somewhat carried away with a nationalistic sense of superiority (he argued that Cro-Magnon people still lived in parts of Norway etc.) [61].

#### Martin P:son Nilsson

Nilsson was the professor of ancient Greek, classical archeology and ancient history at Lund. His two contributions to Hereditas the first in 1921 and the second one in 1939 [62, 63] represent a remarkable interdisciplinary approach intended to give a genetic explanation to history. Natural sciences and humanities are often seen to represent two separate cultures, characterized by mutual misunderstanding and controversies. How did Nilsson bridge history and genetics at a time when the learned discourse underwent a dramatic change?

In 1921 Nilsson [62] explains the fall of Rome as a result of unfettered bastardizing between races. He was certainly acquainted with an immensely influential book of the time. Oswald Spengler argued in Der Untergang des Abendlandes (The decline of the West) that civilizations are superorganisms each with a predictable and limited lifespan. The West (i.e. the Greco-Roman civilization) had fallen once and the second fall is coming near. Spengler had used the word “race” copiously, but not in a biological sense. As told above, Lundborg and Mjøen had promoted the deleterious biological effect of race mixing.

Bengtsson [63] and I [37] have described the contents of Nilsson [62] so that a short rehearsal only will suffice here. Inbreeding within a race was the rule in olden times. Later on, Roman upper classes did not propagate their kind fast enough with the result that the scum of the earth populated the capital. The empire gave rise to unlimited bastardizing; mixing races lowers the fitness of the better one. Hybrids may have been intelligent but they lacked moral stamina. The result was chaos. Evidently Nilsson had read Lundborg [54, 55] who used an identical terminology in a medical context. Herman Nilsson-Ehle a friend of Nilsson [64], evidently told him what was newest new in Hereditas. Nilsson expanded the above ideas in a booklet published by the Society for Racial Biology [65].

Nilsson’s second contribution to Hereditas [63] is an about-face [37]. He has clearly intended to write an essay rather than a scientific treatise. He points out that dog breeders mix races to obtain desirable characteristics. Nilsson notes briefly that he [62] had argued that the chaos of non-complementary genes brought down the Roman Empire.

Here Nilsson demonstrates that the ancient civilizations were created and borne by mixed peoples. His examples include the Egyptians, Babylonians, Assyrians, Greeks, citizens of the Roman Republic etc. The Jews, in particular, are a gifted people with multiple origins. An initial chaos will give rise to a number of biotypes: some are definitely positive and persist while some others will disappear in the course of genetic segregation and selection. Indo-Europeans were a destructive element that was domesticated through interbreeding with the older Europeans. All peoples of Europe and the Near East descend from this process.

Nilsson was an outsider in a genetics journal. This gave him an opportunity to see how times and values had changed in the span of less than twenty years. In 1921 he [62] wrote at a time when physical race biology was cutting-edge science, backed through a political consensus. Sterilization laws would follow in due time. As we shall see, eugenics changed direction in the 1930’s from sterilization towards a social policy of the welfare state. Anthropological race biology was increasingly seen as an aberration. Hitler’s rise to power had led to vulgar and unrestrained doctrines of race that in the Nordic countries were seen to represent a travesty of science. Nilsson’s essay of 1939 [63] was printed in the June of the last summer of peace in the long shadow of the impending war that would engulf four of the five Nordic countries.

#### Søren Hansen

Hansen was a leading Danish anthropologist and a founding member of the Permanent International Eugenics Committee. He had a single contribution to Hereditas in 1922 [66], a study on the inheritance of dementia praecox (schizophrenia). He uses European royal houses to illustrate the contribution of heredity vs. environment and ponders the plausibility of different alternatives. It represents human genetics pure and simple without eugenic overtones.

#### Kaarlo Hildén

Hildén was the professor of geography at the Helsinki School of Economics. His credentials as an anthropologist included an expedition to the Altai mountains in Siberia. In 1921 he undertook to study the isolated and inbred Swedish speaking population on the island of Ruhnu (*Runö*), about equidistant between Estonia and Latvia in the Gulf of Riga. Using observations and church records he analyzed the inheritance of the shape of the earlobe Hildén [67], head dimensions [68] and the shape of the nose [69]. These three papers in Hereditas describe the inheritance of characters relevant to anthropometrics.

#### Göte Turesson

Göte Turesson is certainly one of the foremost authors in Hereditas. The reason that he here has fallen among human race biologists is certainly not his making. Turesson made truly pioneering contributions to ecological genetics, published in Hereditas. Herman Nilsson-Ehle supervised his PhD study at Åkarp and Lund. Turesson’s basic approach is seen in Erwin Baur’s influential textbook [70]. Baur was a friend of Nilsson-Ehle and Scandinavian nature.

Turesson observed variation in common plants with different breeding systems both in the wild and under cultivation, including transplantation experiments. Some plants remained constant while some responded to the new environment. He partitioned the variation into a phenotypic and genotypic component and he coined in 1922 the term *ecotype* to describe the genotypic response to the habitat [71].

The reason why I take up Turesson in the company of race biologists is that he furthered typological thinking to biology and Hereditas at a time when population genetics was on the rise among human geneticists. Nilsson’s [61] and Lundborg’s [55, 56] reasoning comes close to Turesson’s. Turesson had, however, based his conclusions on experimental evidence and on plants. Unlike animals, plants control the process of fertilization through breeding systems, which range from complete apomixis (each plant makes identical copies or *clones* of itself) to complete outbreeding imposed by dioicy or self-incompatibility systems. Population biologists have often difficulties in understanding that plants need not behave as members of an ideal population.

#### Kristine Bonnevie

In 1912 Bonnevie was appointed professor of zoology at the University of Kristiania (Oslo). She had studied widely abroad, namely in the laboratories of Arnold Lang in Zürich, Theodor Boveri in Würzburg and Edmund Wilson in New York. It is difficult to imagine more outstanding hosts. Lang hed developed cytological techniques, Boveri and Wilson’s student Sutton showed that genes reside in chromosomes. Bonnevie specialized in the cytology of marine invertebrates. In 1916 she founded the Institute for Genetic Research to study human genetics at the University of Kristiania [72]. Bonnevie collaborated with the Drosophila geneticist Otto Lous Mohr, the professor of anatomy.

Bonnevie had three papers in Hereditas [73–75]. She quantified the differences underlying fingerprint patterns and attempted to determine how they are inherited. In the second paper she tried to find out whether the fingerprint pattern was associated with IQ among schoolchildren. The patterns were used in the diagnosis of metal deficiency. She noted that fingerprints had been used in ethnographic contexts but did not take up race biology. The third is about the relation of left-handedness to fingerprints.

#### Gert Bonnier

A Drosophila geneticist, Bonnier studied theoretical aspects of human genetics; something that may have helped him to establish the animal breeding institute at Wiad in the 1930’s. He was the first professor of genetics of the University of Stockholm.

Bonnier [76] criticized in *Acta Zoologica* (the journal of Stockholm zoologists) that the association of heterozygosity and TBC proposed by Lundborg in 1920 and 1921 [55, 56] in Hereditas. In Hereditas he used French, the language of mathematics, in defining the application of statistics in biology [77]; a theme that he developed later on in a textbook. Even though human cytogenetics was at an elementary stage in the 1930’s, he described a case of heritable sex-mosaicism in Hereditas [78]. Professor Solomon Levit of Moscow criticized his statistics and consequently Bonnier [79] published an apology and correction. This did not reach Dr. Levit; Lysenko had smeared him as traitor and he was shot in early 1938.

#### Gunnar Dahlberg

Dahlberg had studied medicine at Uppsala. As mentioned earlier, he was appointed Lundborg’s assistant at the State Institute for racial biology in 1922 and, in 1935, was elected Lundborg’s successor as the head of the institute. He understood that both statistics and population genetics showed the hollowness of race biology. Consequently, he changed the name of the Institute to one of human genetics and racial biology.

In his first paper in Hereditas in 1923 Dahlberg [80] used statistics to differentiate between mono- and dizygotic twins in two extensive data sets, one collected from Germany by Weinberg and the other by Bonnevie in Norway.

In 1930 Dahlberg had two papers in Hereditas [81, 82]. The first introduced a new statistical method in treating family data. It drew forth a response from Wilhelm Weinberg [53, 83] in Hereditas. His second paper [82] is an analysis on the effect of inbreeding in different modes of inheritance. Dahlberg showed here, as in his later papers in Hereditas [84] a good grasp of both statistics and population genetics.

#### Victor Berglund

Berglund was a Swedish physician, painter and sculptor had a contribution on the occurrence of hypotrichosis (weak hair growth) within a family that was published posthumously in 1924 [85].

#### Jon Alfred Mjøen

As stated above, the eugenicist and race biologist Mjøen may not have earned kudos as a geneticist, but he succeeded in having papers published not only in Hereditas but also in e.g. Zeitschrift für induktive Abstammungs- und Vererbungslehre, in spite of,or rather because of, the rather unusual subject, namely the inheritance of musical talent [86]. He used multiple approaches in the study: he sent questionnaires wide and far, observed directly the level of perception and assessed the contribution of the parents on musical talent in large families. The results were straightforward. Untalented parents have seldom talented children, while talented parents never have untalented offspring. Fritjof Mjöen, J.A. Mjøen’s son, extended these observations to include the perception of pitch [87].

#### Lännart Ribbing

Ribbing, a specialist in the comparative anatomy of vertebrates, made an entry to race biology: he took measures on Icelanders. He was a member of first executive board of the Mendelian Society and presided at the first meetings [16]. In 1922 he was appointed professor of anatomy at the Royal Swedish Academy of Fine Arts. In addition, Ribbing won acclaim as a poet. He was one of the few who published in French in Hereditas.

Ribbing had a single contribution to Hereditas [88]. It represents skull measurement or craniology, based on three skulls dug out of the earth at Vellinge in southwestern Scania. The oldest of the skulls hails from Stone Age, the next from Iron Age while the third is modern. This modest sample of three is meticulously photographed and all pertinent parameters are given.

Why was a contribution like this included in a journal otherwise dedicated to the branch of science founded by Friar Gregor Mendel? Ribbing argues that the skulls are remarkably similar. Consequently, a morphologically uniform human population has inhabited Vellinge since the days of the first one: “an intelligent person representing a distinguished culture” [88].

#### Sven Wahlund

Wahlund, a statistician at the State Institute for Racial Biology at Uppsala, published with Lundborg and Dahlberg on Sámi [42, 43]) but he had papers on plant breeding in Hereditas as well. He is the author of one of the truly seminal papers ever published in Hereditas: Wahlund 1928 [89], in which he describes the *Wahlund effect*, an ingredient in standard texts on population genetics. This arguably most famous Swedish contribution to population genetics states that as long as there is allele frequency variation among subdivisions of a population, the genotype frequencies of the population will exhibit a deficiency of heterozygotes and an excess of homozygotes relative to Hardy-Weinberg expectations.

In 1929 Wahlund [90] demonstrated a potential use for the enormous amount of data stored at the Institute for Racial Biology. Lundborg and Linders [34] had argued that anthropological measures are associated with social strata; the ones better off are taller and have a higher cephalic index than the population at large, while German authors seemed to have found evidence that the “Germanic” characteristics are selected against in cities. Using a sample of 677 students in among the 47.887 conscripts arranged in order by Lundborg & Linders [34], Wahlund compared the means of different body parts among students and the general population of Sweden. The students were, on an average, 3 cm taller and differed significantly also from their compatriots of the same age in the width of the zygomatic arch etc. Interestingly, the results were neither correlated to the geographical origin of the students nor to the social status of their parents.

Wahlund notes that the results are apparently associated with the upward social mobility of the students. What is cause and what is effect is left open. Wahlund warns against generalizing the results to other populations; there is simply no reference material anywhere.

#### Oluf Thomsen

Thomsen was the professor of pathology at the University of Copenhagen. He was the driving force in the establishment of the Department of Human genetics at Copenhagen. His first contribution to Hereditas [91] was a description of a hereditary growth anomaly of the thumb. He shifted his attention to the inheritance of blood groups [92, 93] and several others.

Thomsen wrote popular presentations of genetics. They had titles such as Human heredity (*Menneskets Arvelighedsforhold*). The first editions contained much eugenics. His outlook changed as years passed and he was actively studying blood groups and he became acquainted with population genetics. An edition of 1934 [94] has chapters on eugenics and human races. As for eugenics he points out that there is no need to do anything about dominant traits, since they will take care of themselves. Anything with recessive traits will be ineffective. Thomsen advocates “passivity” in the enforcement of sterilization laws. He downplays races; points out that they are not uniform with regard to any alleles etc. He shows that the Jews are not a race and that the races of Europe are, if anything, mixed.

#### Torsten Sjögren

Sjögren was employed at the Institute for Racial Biology in 1925. He had an extensive treatise on Tay-Sachs disease ((juvenile amaurotic idiocy) in 1931 in Hereditas [95] as well as a contribution to the Eighth International Congress of Genetics in 1949 [96]. He was a serious contender for the chair of director vacated by Lundborg. A psychiatrist, he believed in the efficacy of sterilization already in childhood [17]. He has gained some notoriety as a member of eugenic organizations that had a racist tinge [97]. Sjögren was the professor of psychiatry at Karolinska Institutet in Stockholm from 1945 to 1961.

#### Thordar Quelprud

Quelprud, a student of Bonnevie, described a Drosophila mutant in Hereditas. He studied anatomy in Germany, where he became a naive national socialist Even though he was not a PhD, the Quisling administration made him docent in 1942 and he created a Department of Hereditary Biology at the University of Oslo, located between the premises of Bonnevie and the anatomist and anthropologist Emil Kristian Schreiner (who had done time at the Grini concentration camp). The new department folded when the university was shut down in late 1943 [98].

#### Jan Arvid Böök

Böök moved from Lund to Uppsala, where he was appointed head of the State Institute of Medical Genetics and Racial Biology in 1957 following Dahlberg’s retirement. As told above, Dahlberg had directed the State Institute to one of human genetics away from the race biology of Lundborg. Böök cut the last ties to race biology: in 1959 the State Institute was incorporated into the University of Uppsala as the Department of Human Genetics [99].

Böök had two papers in Hereditas in 1948 [100, 101]. Both deal with the effect of cousin marriages, so that the latter takes up the inheritance of clubfoot, including the mutation rate and twin studies.

#### The Eighth International Congress of Genetics

The Stockholm International Congress of Genetics was held in 1948. The proceedings produced a flurry of papers, published as supplement 35S in Hereditas a year later. There is a single paper on anthropological race biology: Corrado Gini of Rome presented a treatise on the physical assimilation of Albanians to a host population in Calabria, Italy [102]. The Calabrians were short and dolichocephalic, while the Albanians were tall and brachycephalic. Gini gives comparisons among several other physical characteristics as well.

There were four non-Nordic papers on human genetics, namely JBS Haldane on the mutation rate of human genes [103], Lionel Sharples Penrose on palmar patterns in relation to mongolism (Down’s syndrome) [104], Laurence H. Snyder on linkage relationships of sickle-cell anemia [105] and Alexander S. Wiener on Rh blood types [106].

#### Tage Kemp

Tage Kemp studied medicine at Copenhagen with Oluf Thomsen as supervisor. He visited Charles Davenport’s Eugenics Record Office at Cold Spring Harbor. Davenport was the leader of the American eugenics movement. In 1932 Kemp participated in the Third International Eugenics Congress in New York with a paper on prostitution and heredity. He studied chromosomes and mouse genetics and had a paper on the latter subject in Hereditas. The University Institute of Human Genetics and Eugenics was established in 1938 on the initiative of Oluf Thomsen. He had contacted the Rockefeller Foundation in 1934 and obtained 250.000 Danish Crowns + 110.000 Crowns as a grant, the interest of which would be used to maintain the institute. The Danish state would be responsible for the personnel and day-to-day costs. Kemp was the head of the institute from 1938 on; ten years later he was installed as professor of genetics and eugenics at the Medical Faculty of the University. Kemp abhorred the propagandizing and pseudoscientific Nazi race hygiene. He even started criticizing eugenics, a stance recommended by the Rockefeller Foundation in the late 1930’s [11].

Kemp had a contribution to the Eighth International Congress of Genetics in Stockholm titled “The rise of human genetics” [107]. It is a short history of the subject with a particular emphasis on the development in Copenhagen.

#### Nils von Hofsten

As told above, von Hofsten had the task of reviewing the applications for sterilization that were sent to the State Medical Board of Sweden. He was mainly responsible for the new law of 1941. He evidently never did original research on genetics but wrote popular papers on sterilization in Swedish in the 1930’s and 1940’s. He had written a textbook of genetics in 1919 that (in spite of some howling errors) is a balanced treatment [108]. As an outsider, he had no ax to grind. He presented eugenic literature and warned that the books of Mjøen and, in particular, the British eugenicist Caleb W. Saleeby, should be read critically. In addition, von Hofsten criticized the extreme views of American eugenicists.

von Hofsten had one paper on the subject of eugenics in 1951 in Hereditas [109]: “The genetic effect of negative selection in man”. The paper extends over more than 100 pages of curves, graphs and illustrations. In effect it is a rehearsal. To give an example, HTJ Norton had worked much of it out in an appendix to a book on mimicry in butterflies in 1915 [110]. In retrospect, one wonders why this overlong paper was ever written. Von Hofsten had seen that “the sterilization policy had been the subject of a lively interest at the International Congress of Genetics in 1948”. Consequently, he wished to demonstrate that sterilization had a firm scientific foundation: genetic ills can express themselves in many ways and one must know how to get rid of them.

#### Arne Müntzing

Müntzing has a secure place in Hereditas’ Hall of Fame. In the 1930’s he published a series of papers in our journal that were certainly ground-breaking, e.g. [111]. He had crossed two diploid hemp-nettles, *Galeopsis speciosa* with *G. pubescens* and obtained tetraploid offspring that clearly represented a well-known tetraploid taxon, namely *G. tetrahit*. In short, Müntzing had shown how a plant species had originated. He became the respected doyen of Nordic genetics for years to come, not least as the editor-in-chief of Hereditas through 1955–1977.

Müntzing wrote an influential textbook of genetics, Genetic Research (*Ärftlighetsforsking*). It went through ten editions and was translated into several languages. The book is strong on cytogenetics as well as plant and animal breeding. It has photos of Europeans belonging to different races presented in the traditional typological fashion, including the Sámi as a kind of outgroup. Müntzing condemned racism in no uncertain terms. He changed eugenics of the first editions to genetic counseling later on. Mogens Westergaard of Copenhagen published also in 1953 a general textbook of genetics in Danish: *Arvelighedslæren*. He shared the general outlook with Müntzing and included some race biology [112].

## Conclusions

Biological anthropology was effectively dead as a branch of genetics in the late 1930’s. To mention an example, in the US Franz Boas and his students showed that race categories were overlapping, ill-defined and historically inconsistent. They showed that the head form of immigrants changed from one generation to the next with changed conditions of life; in other words it was subject to the influence of the environment [112]. The horrors of the Second World War finished off what little may have remained. The First International Congress of Human Genetics held in Copenhagen in 1956 did not mention things such as race, or nation, the topic of interest was *the human race* or mankind.

Laws allowing sterilization on eugenic grounds were enacted in Denmark, Finland, Iceland, Norway and Sweden in a course of a few years in the 1930’s. The sterilization policies are now seen as a major human tragedy: compensation is paid to its victims. Interestigly, there is only a single paper discussing the justification of negative eugenics in Hereditas [109]. It represents a belated defense of a policy that had lost its adherents within the community of Nordic geneticists.

In the 1950’s the university departments changed names. Human genetics had an advisory function that guided the work of hospitals and doctors. It took some 15 years before the sterilization laws were abolished. This put an end to this advisory function as well. It had already earlier been supplanted by prenatal diagnosis and a research program differentiated to cover a multitude of genetic diseases.

As mentioned in the beginning, race is now hardly a concern for scientists [1]). It remains, however, a virtually explosive political issue. August names such as Linnaeus and Ronald A. Fisher are removed from public consciousness [113] for their sins. The former had attributed negative characteristics to nonwhite populations and the latter had championed eugenics.

Hereditas was envisioned to treat race biology as a major branch of applied genetics, on par with plant breeding in importance. The contents of Hereditas show how the ideas of race gave way to human genetics. Hereditas has a long record of publishing milestones in human genetics such as the demonstration of the human chromosome number by Tjio and Levan [114] and the identification of all human chromosomes [115].
